# Dynamic viscoelastic characterisation of human osteochondral tissue: understanding the effect of the cartilage-bone interface

**DOI:** 10.1186/s12891-019-2959-4

**Published:** 2019-11-30

**Authors:** Sophie E. Mountcastle, Piers Allen, Ben O. L. Mellors, Bernard M. Lawless, Megan E. Cooke, Carolina E. Lavecchia, Natasha L. A. Fell, Daniel M. Espino, Simon W. Jones, Sophie C. Cox

**Affiliations:** 1 EPSRC Centre for Doctoral Training in Physical Sciences for Health, University of Birmingham, Edgbaston, Birmingham, B15 2TT UK; 2 School of Chemical Engineering, College of Engineering and Physical Sciences, University of Birmingham, Edgbaston, Birmingham, B15 2TT UK; 3 Department of Mechanical Engineering, University of Birmingham, Edgbaston, Birmingham, B15 2TT UK; 4 Centre for Musculoskeletal Ageing Research, Queen Elizabeth Hospital, University of Birmingham, Edgbaston, Birmingham, B15 2TT UK

**Keywords:** Articular cartilage, Dynamic mechanical analysis, Osteoarthritis, Subchondral bone, Viscoelasticity

## Abstract

**Background:**

Despite it being known that subchondral bone affects the viscoelasticity of cartilage, there has been little research into the mechanical properties of osteochondral tissue as a whole system. This study aims to unearth new knowledge concerning the dynamic behaviour of human subchondral bone and how energy is transferred through the cartilage-bone interface.

**Methods:**

Dynamic mechanical analysis was used to determine the frequency-dependent (1–90 Hz) viscoelastic properties of the osteochondral unit (cartilage-bone system) as well as isolated cartilage and bone specimens extracted from human femoral heads obtained from patients undergoing total hip replacement surgery, with a mean age of 78 years (*N* = 5, *n* = 22). Bone mineral density (BMD) was also determined for samples using micro-computed tomography as a marker of tissue health.

**Results:**

Cartilage storage and loss moduli along with bone storage modulus were found to increase logarithmically (*p* < 0.05) with frequency. The mean cartilage storage modulus was 34.4 ± 3.35 MPa and loss modulus was 6.17 ± 0.48 MPa (mean ± standard deviation). In contrast, bone loss modulus decreased logarithmically between 1 and 90 Hz (*p* < 0.05). The storage stiffness of the cartilage-bone-core was found to be frequency-dependent with a mean value of 1016 ± 54.0 N.mm^− 1^, while the loss stiffness was determined to be frequency-independent at 78.84 ± 2.48 N.mm^− 1^. Notably, a statistically significant (*p* < 0.05) linear correlation was found between the total energy dissipated from the isolated cartilage specimens, and the BMD of the isolated bone specimens at all frequencies except at 90 Hz (*p* = 0.09).

**Conclusions:**

The viscoelastic properties of the cartilage-bone core were significantly different to the tissues in isolation (*p* < 0.05). Results from this study demonstrate that the functionality of these tissues arises because they operate as a unit. This is evidenced through the link between cartilage energy dissipated and bone BMD. The results may provide insights into the functionality of the osteochondral unit, which may offer further understanding of disease progression, such as osteoarthritis (OA). Furthermore, the results emphasise the importance of studying human tissue, as bovine models do not always display the same trends.

## Background

The cartilage-bone interface in articulating joints is key to moderating the transmission of tensile, compressive, and shear forces from the articular cartilage to the subchondral bone [1]. The complex organisation of collagen fibres within cartilage, in part, enables it to store and dissipate energy [2], and articular cartilage is considered to be a frequency-dependent viscoelastic structure [3–6]. Studies that have analysed this interface have primarily focused on its structure and composition, characterising the calcified cartilage and underlying tidemark where collagen type I and II integrate [7–10]. More recently, biological signalling between articular cartilage and subchondral bone have been identified through vascular microchannels that traverse the subchondral bone and calcified cartilage, allowing diffusion of small molecules [11].

The viscoelastic properties of isolated articular cartilage have been well-characterised. Edelsten et al. demonstrated that cartilage behaves non-linearly under high-speed loading [12]. More recently, Lawless et al. [13] focused on observations of isolated cartilage under load at frequencies across a physiological range (1–92 Hz [3]) and determined that the storage and loss moduli were frequency-dependent. Despite providing valuable results on the behaviour of articular cartilage under dynamic loading, both these studies looked at cartilage in isolation and therefore were not able to offer insight on the behaviour of the cartilage-bone system as a whole.

In the last decade, it has been shown that severe impacts to articulating joints are known to result in damage to the bone rather than the cartilage [14]. High-impact loading has been suggested as a major risk factor of osteoarthritis (OA), and subchondral bone has been identified as having a role in the progression of the disease [15–17]. While it is known that both subchondral bone and loading frequency have a significant effect on cartilage viscoelasticity [13, 18], there is currently a gap in our understanding of subchondral bone’s response to loading, independent of cartilage. Recent work by Fell et al. endeavoured to rectify this by conducting an analysis of the mechanical properties of bovine cartilage and bone [19]. Interestingly, a linear correlation between bone mineral density (BMD) and cartilage viscoelasticity was identified, suggesting there is an important relationship between the two tissues at the interface. However, to date, there are no studies of this nature conducted in human tissue.

The mechanical properties of osteochondral tissues are evidently very complex. In addition to the studies previously described that have measured cartilage viscoelasticity directly, mathematical models have been developed to elucidate further information on how articular cartilage functions in vivo. For example, local temperature changes in cartilage under load have been assessed through the development of a model to determine temperature increase from cartilage intrinsic viscoelasticity [20]. Modelling cartilage allows for properties that are difficult to measure directly in vivo to be examined. However, there are disputes regarding some mathematical models of cartilage. For instance, as proposed by Huyghe et al. [21], triphasic theory may not respect the laws of thermodynamics. Given the complexity of both tissues, studying and modelling cartilage or bone in isolation does not provide a complete picture of either tissue’s capacity to store and dissipate energy [19]. Whilst there are studies that have identified the mechanical properties of animal cartilage, both on and off bone [13, 18], the role of the cartilage-bone interface in the dissipation of energy has not previously been investigated in human tissue, nor has it been incorporated into mathematical models of articular cartilage.

The aim of this study is to characterise the viscoelastic properties of human osteochondral tissues and assess the dissipation of energy by these tissues. More specifically, an approach that characterises viscoelastic behaviour of the osteochondral core and isolated tissues in a physiological frequency range has advantages in being able to assess the significance of the interactions between the two tissues. Therefore, energy dissipation has been analysed for osteochondral tissues. By using dynamic mechanical analysis (DMA), the viscoelastic properties of the human cartilage-bone unit were directly compared to the subchondral bone and articular cartilage. Furthermore, the bone mineral density (BMD) of the subchondral bone was determined, by micro-computed tomography (μ-CT), to identify any relationships with its mechanical properties, or the viscoelastic properties of cartilage.

## Methods

### Specimen preparation

**Fig. 1:**
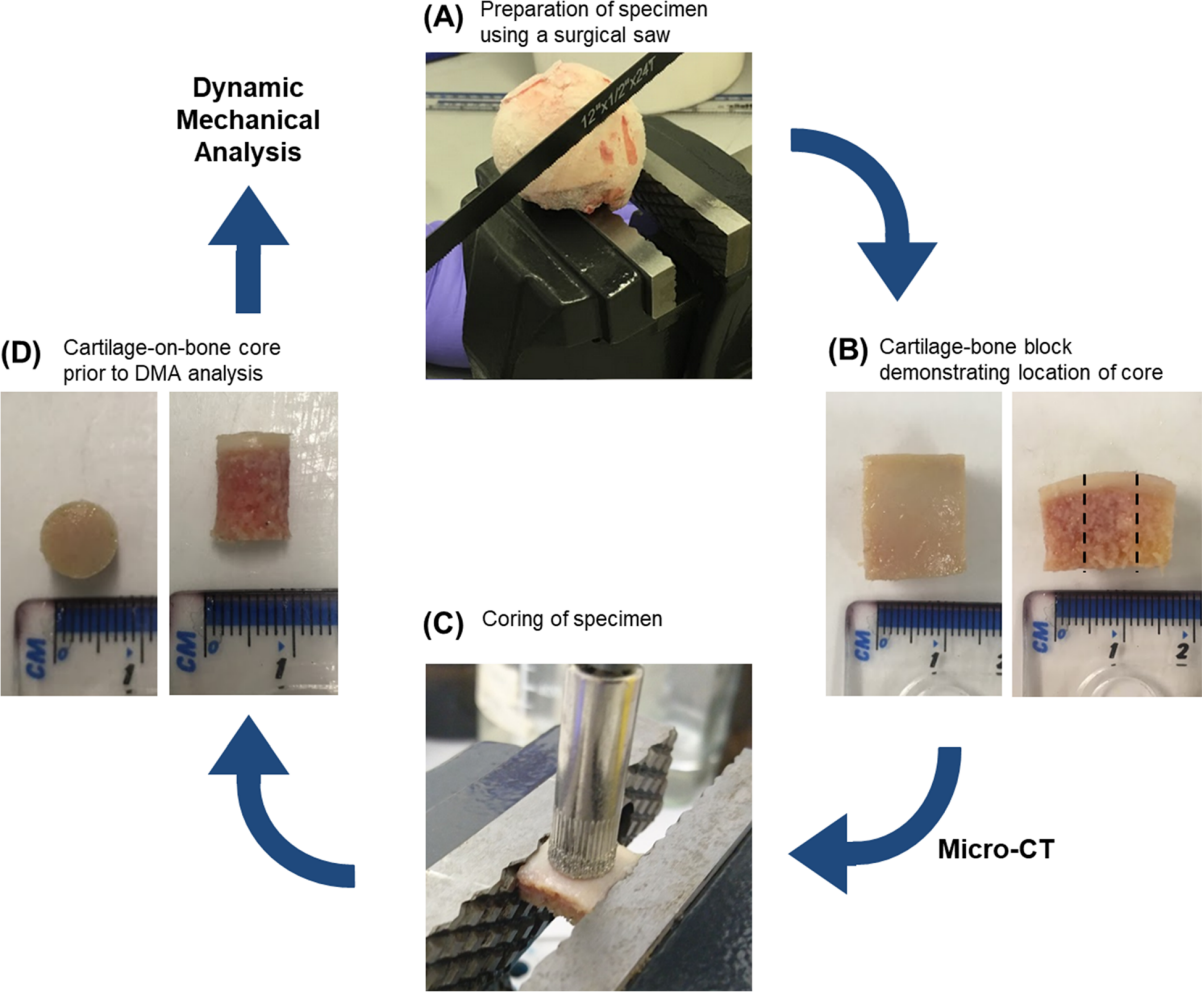
Flow diagram illustrating femoral head specimen preparation and coring: **a** Preparation of specimen using a surgical saw, **b** Example of cartilage-bone block prior to μ-CT analysis demonstrating where core was taken, **c** Coring of specimen, and **d** Example of cartilage-bone core prior to dynamic mechanical analysis

Femoral heads (*N* = 5) (Table 1) were obtained from patients undergoing total hip replacement surgery following fracture of the neck of femur. Patients had no reported history of joint pain or OA disease prior to fracture of the femoral neck. Furthermore, chondropathy assessment of femoral head cartilage integrity revealed the absence of any cartilage lesions or erosions that might have indicated signs of OA damage. Thus, the cartilage was deemed to be healthy. Ethical approval was provided by the United Kingdom National Research Ethics Service (East of Scotland Research Ethics Service, 11/ES/1044) and consent for the use of their tissue for research was given by the patients. Upon arrival, specimens were stored at -80 °C, which has been previously shown not to affect the viscoelastic properties of the specimens [22]. While each specimen was frozen, a total of 22 cartilage-bone blocks approximately 14 × 14 mm in area (*n* = 22) were obtained using a surgical saw (Fig. 1a). The depth of the block varied depending on the specimen. Prior to testing, the specimens were macroscopically examined, and only intact specimens were thawed in Ringer’s solution at 4 °C overnight [13].

**Table 1:** Specimen information

Specimen Name	L/R Hip	Age	Gender	Weight
RHH214	R	76	M	OW
RHH217	R	72	F	NW
RHH220	L	85	M	NW
RHH238	L	85	F	OW
RHH239	R	71	M	NW

*R* Right, *L* Left, *M* Male, *F* Female, *OW* Overweight, *NW* Normal Weight

Weights categorised from patient BMI: 18.5 to 24.9 = NW, 25 to 29.9 = OW

### Micro-computed tomography (μ-CT)

Specimens were scanned as 14 × 14 mm blocks to prevent swarf created during sample sectioning from affecting the subsequent analysis of μ-CT data. Specimens were secured in a low X-ray attenuation tube and individually scanned using a Skyscan 1172 scanner (Bruker Micro-CT, Belgium). A 180° scan was performed with 80 kV maximum X-ray energy and 8 W beam power, using an aluminium and copper filter, with a pixel size of 12.03 μm. The data was reconstructed using NRecon V1.6.10.2 (Bruker Micro-CT, Belgium) using a beam hardening correction of 30%, a ring artefact correction of 4.0, and a smoothing value of 2.0. Bone mineral density (BMD) was calibrated from the attenuation coefficient using CT-analyser software V1.15.4.0 (CTAn) (Bruker Micro-CT, Belgium). Two phantom rods made up of epoxy resin with embedded fine calcium hydroxylapatite (CaHA) powder at concentrations of 0.25 and 0.75 g.cm^− 3^ were scanned and reconstructed using the same parameters as used for the human specimens. BMD values of a cylindrical volume of interest the same size as the sample used for subsequent mechanical analysis (8 mm diameter) for each reconstructed dataset were calculated according to a standard method [23].

### Specimen coring

Following thawing, sectioning of the specimen, and μ-CT scanning, a core 8 mm in diameter was taken from each specimen using a pillar drill with a diamond-coated drill bit (Fig. 1c). After harvesting this cartilage-bone core, the specimen was hydrated in Ringer’s solution for 30 min, as per previous studies [4, 5]. Samples were reviewed for macroscopic damage after coring and only samples with intact surfaces were tested, as surface cracks alter the mechanical properties of cartilage. The effect of the curvature evident in the cartilage-bone block (Fig. 1b) was considered to be negligible once a core was taken (Fig. 1d) and therefore not taken into account in subsequent analyses in this study.

### Dynamic mechanical analysis (DMA)

Dynamic Mechanical Analysis (DMA) subjects a specimen to a sinusoidal load and measures its out-of-phase displacement response [24]. This enables the calculation of a structure, or a material’s, viscoelastic properties [25]. The viscoelastic properties of all specimens were determined using a Bose ElectroForce 3200 with WinTest 4.1 software (Bose ElectroForce Group, New Castle, Delaware, USA, now TA Instruments). This method has been previously used to determine the viscoelastic properties of bovine and human cartilage [4, 19, 26], and bovine cartilage on bone [6, 13, 18, 19, 27].

By using a cylindrical compression platen (20 mm diameter) under unconfined conditions, a sinusoidally compressive load ranging between 37.7–85.5 N was applied to all specimens, following a preload of 4 N. This induced a stress range between 0.75–1.7 MPa, as 1.7 MPa is estimated cartilage stress during walking [27]. All specimens were tested in air at room temperature; as results in literature suggest that dehydration should not occur over the short duration of each frequency-sweep [4]. The sinusoidal force was applied using a frequency-sweep of: 1, 8, 10, 12, 30, 50, 70, and 90 Hz. The specimens were subjected to two preload conditions: 25 Hz for 1500 cycles and 50 Hz for 3000 cycles, as cartilage requires application of a series of loading cycles to reach a steady state [6, 28]. After DMA was performed on the osteochondral core, full-thickness cartilage was then removed from the subchondral bone using a scalpel and hydrated in Ringer’s solution for 30 min. Following inspection to ensure there was no damage to the sample, the same DMA procedure as described above was subsequently performed on the isolated subchondral bone and isolated cartilage specimens.

For each frequency sweep conducted, the WinTest DMA software performed a Fourier analysis of the load and displacement sinusoidal waves. From this, the magnitudes of the load (F*), displacement (d*), phase angle (*δ*), and frequency (*f*) were determined, further described elsewhere [25]. The complex stiffness (k*) was then calculated (Eq. 1).

1 $$ {k}^{\ast }=\frac{F^{\ast }}{d^{\ast }} $$

By using the complex stiffness (k*) and phase angle (*δ*), the storage stiffness (*k’*) and loss stiffness (*k”*) were calculated (Eqs. 2 and 3) [25, 29].

2 $$ {k}^{\prime }={k}^{\ast}\left(\cos \delta \right) $$

3 $$ {k}^{\prime \prime }={k}^{\ast }\ \left(\sin \delta \right) $$

Using a shape factor, S, calculated from the diameter (*d*) and height (*h*) of the specimen (Eq. 4), the storage (*E’*) and loss (*E”*) moduli can be determined (Eqs. 5 and 6) [4].

4 $$ S=\frac{\pi {d}^2}{4h} $$

5 $$ {E}^{\prime }=\frac{k^{\ast }\ \cos \delta }{S} $$

6 $$ {E}^{\prime \prime }=\frac{k^{\ast}\sin \delta }{S} $$

*E’* and *E”* were calculated for isolated cartilage and subchondral bone specimens. As the cartilage-bone cores were complex multi-structures, *k’ and k”* were calculated for those specimens. This enabled evaluation of the properties of the overall system with those of the individual tissues. All results are presented against the requested frequency used for DMA for ease of comparison, however actual frequencies measured varied by ±1 Hz.

### Thickness testing

Following DMA, and after removing the cartilage from the bone using a surgical scalpel, the cartilage specimens were hydrated in Ringer’s solution for 30 min, consistent with a previous study [3]. Following hydration, cartilage thickness was determined using an established needle technique accurate to 1 μm resolution [4, 30]. An analogue Vernier calliper was used to calculate the thickness of bone specimens by taking three measurements from each specimen and calculating the mean thickness (resolution of 0.1 mm). Mean average thicknesses of each specimen type can be found in Table 2.

**Table 2:** Specimen thicknesses. Data displayed to 3 significant figures

Specimen type	n number	Mean average thickness (mm)	Standard deviation (mm)
Cartilage-bone core	18	7.43	± 2.17
Cartilage	20	1.48	± 0.43
Bone	18	5.94	± 2.09

### Energy dissipation calculation

**Fig. 2:**
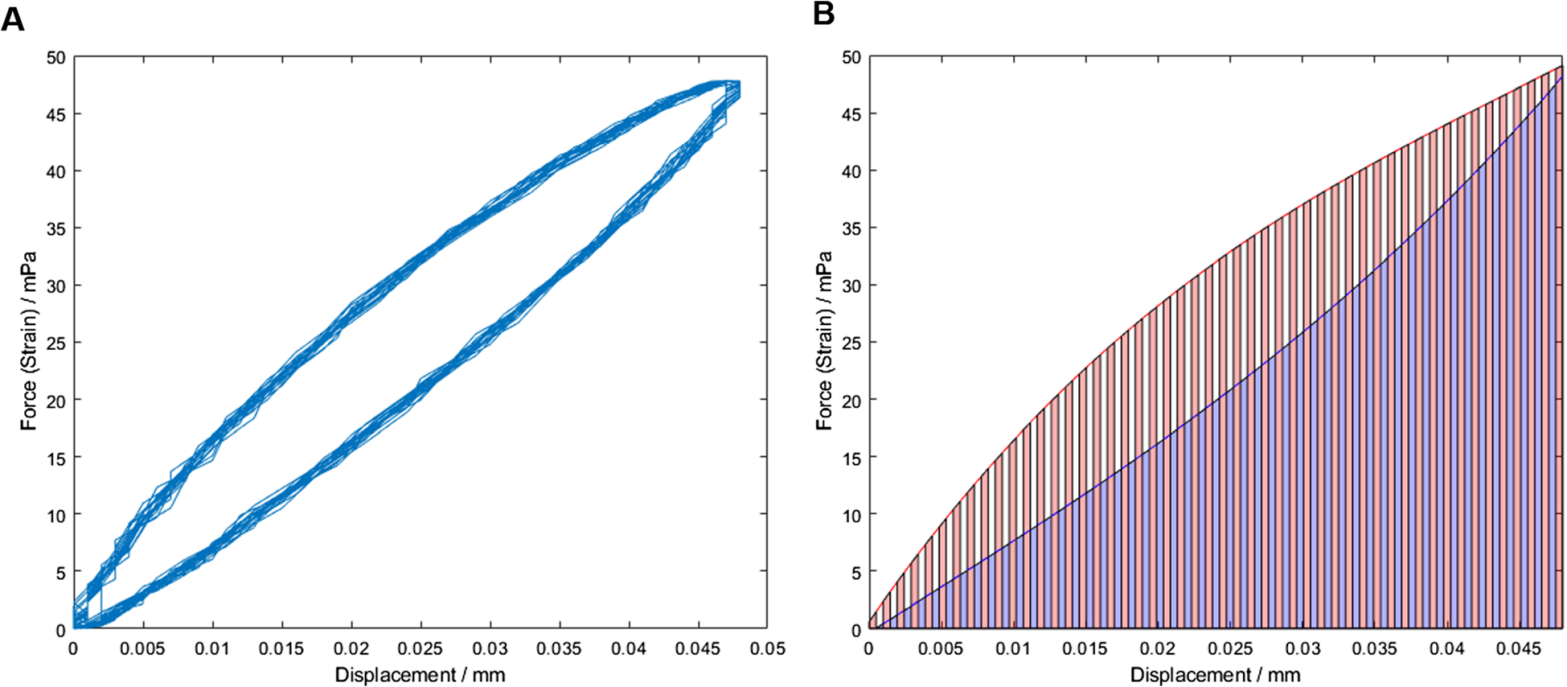
Cartilage hysteresis. Force is given as increase in force during loading cycle. **a** Example of hysteresis loop generated for a single specimen at 30 Hz. **b** Approximation of the area between the upper and lower arcs of each cycle calculated using the trapezoidal rule. The difference between the two areas was determined as the total energy dissipated during that cycle of DMA

Energy dissipation was calculated using Matlab R2018a (Matlab R2018a, MathWorks, Inc., Natick, Massachusetts, USA). Time, force, and displacement data was collected during DMA. Plotting displacement vs force at a given frequency for each specimen produced a hysteresis loop (Fig. 2a). For each complete loop, the area between the arcs was assumed to be the total energy dissipated for that DMA cycle. This was calculated by finding a polynomial approximation of each arc, which was then solved for the range zero to the maximal displacement of that cycle. The area below each polynomial arc was approximated by the trapezoid rule [31] (Fig. 2b); the difference between the two arcs equated to the energy dissipated for that cycle. Finally, the values across all cycles were averaged, resulting in the total energy dissipation per cycle for each specimen at a given frequency.

### Statistical analysis

Statistical analyses were performed using GraphPad Prism 5.03 (GraphPad Software Inc., San Diego, California, USA). 95% confidence intervals were calculated (*N* = 5, *n* = 23); the number of independent patients from which specimens were collected was 5, and between 4 and 5 specimens were measured from each patient. Logarithmic regression curves were fitted to material viscoelastic (*E’* and *E”*) and structure stiffness (*k’* and *k”*) data, where a significant trend was identified (*p* < 0.05) (Eq. 7). Wilcoxon Rank-Sum tests were used to assess whether the cartilage and bone specimens had significantly different material properties (*p* < 0.05). Kruskal-Wallis one-way analysis of variance (ANOVA) tests determined whether structural stiffness variations between the cartilage, bone, and osteochondral cores were significantly different (*p* < 0.05). Tukey’s multiple comparison tests were used to determine the structural stiffness variations that were significantly different between specimen types.

7 $$ {E}^{\prime }=A\ {\log}_e(f)+B\  for\ 1\le f\le 90\  Hz $$

## Results

Of the 23 specimens tested, the results of two were rejected since the cartilage thickness (2.6 and 2.7 mm) lay far outside the normal physiological range of 1–2 mm [32] and may be representative of degenerated cartilage [33]. Peirce’s criterion was used to eliminate the two values [34].

### Viscoelastic properties of isolated cartilage and bone specimens

#### Storage modulus (E’)

**Fig. 3:**
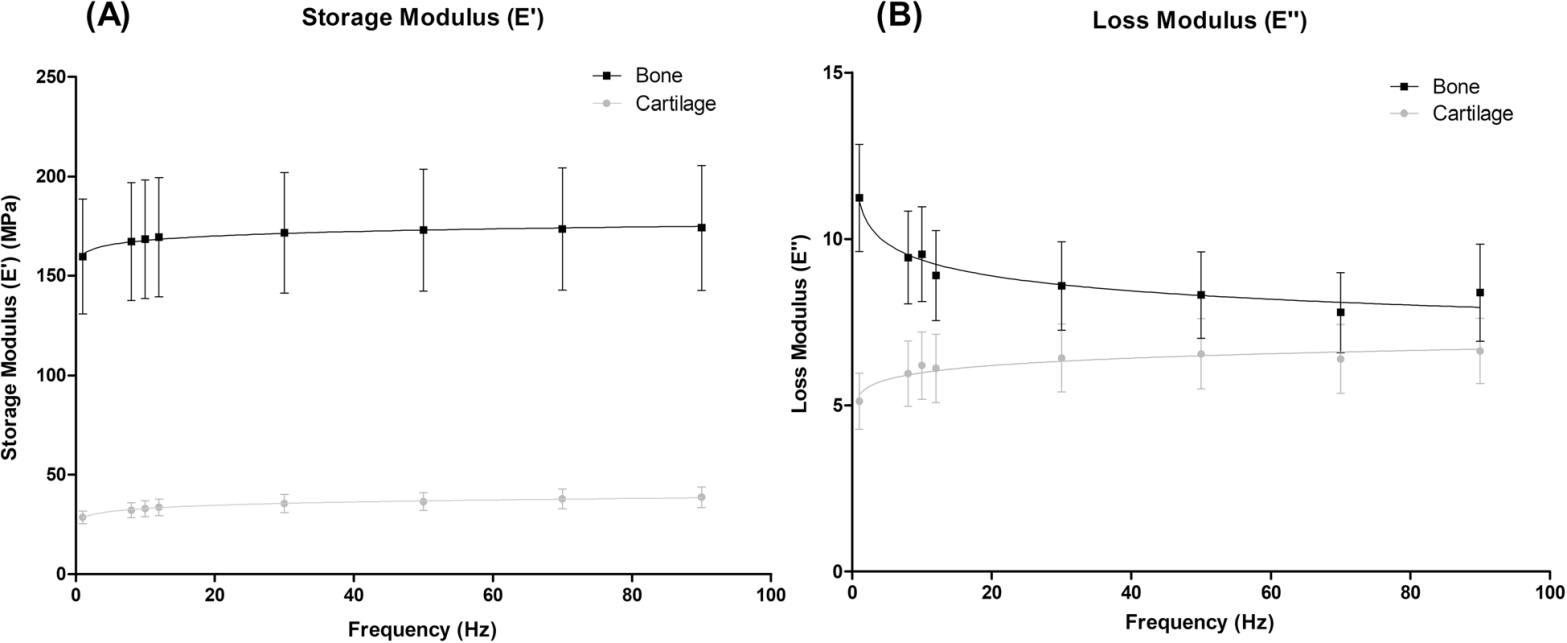
Storage (**a**) and loss (**b**) moduli for isolated cartilage and bone specimens plotted against frequency from 1 to 90 Hz (mean ± 95% confidence intervals, (*N* = 5) with natural logarithmic regression trendlines). In total, 19 cartilage and 18 bone specimens from five femoral heads were tested. Results are displayed on a linear scale

For isolated tissue specimens, *E’* was found to be frequency-dependent and differed significantly (*p* < 0.001) between cartilage and bone specimens (Fig. 3a). The mean value of E’ was 34.4 ± 3.35 MPa for cartilage, while for bone it was considerably higher, 170 ± 4.76 MPa. A logarithmic relationship for *E’* was observed with respect to frequency for both specimens (Eq. 7), with storage modulus increasing with frequency (Fig. 3a).

#### Loss modulus (E”)

Loss modulus was also found to be frequency-dependent and differed significantly (*p* < 0.001) between cartilage and bone specimens (Fig. 3b). A logarithmic relationship for both specimens was observed with respect to frequency (Eq. 7). For cartilage, the mean *E”* was 6.17 ± 0.48 MPa and for bone, *E”* was 9.02 ± 1.07 MPa. The loss modulus for cartilage increased with increasing frequency, while for bone it decreased with increasing frequency (Fig. 3b).

### Viscoelastic properties of cartilage-bone cores

#### Storage stiffness (k’)

**Fig. 4:**
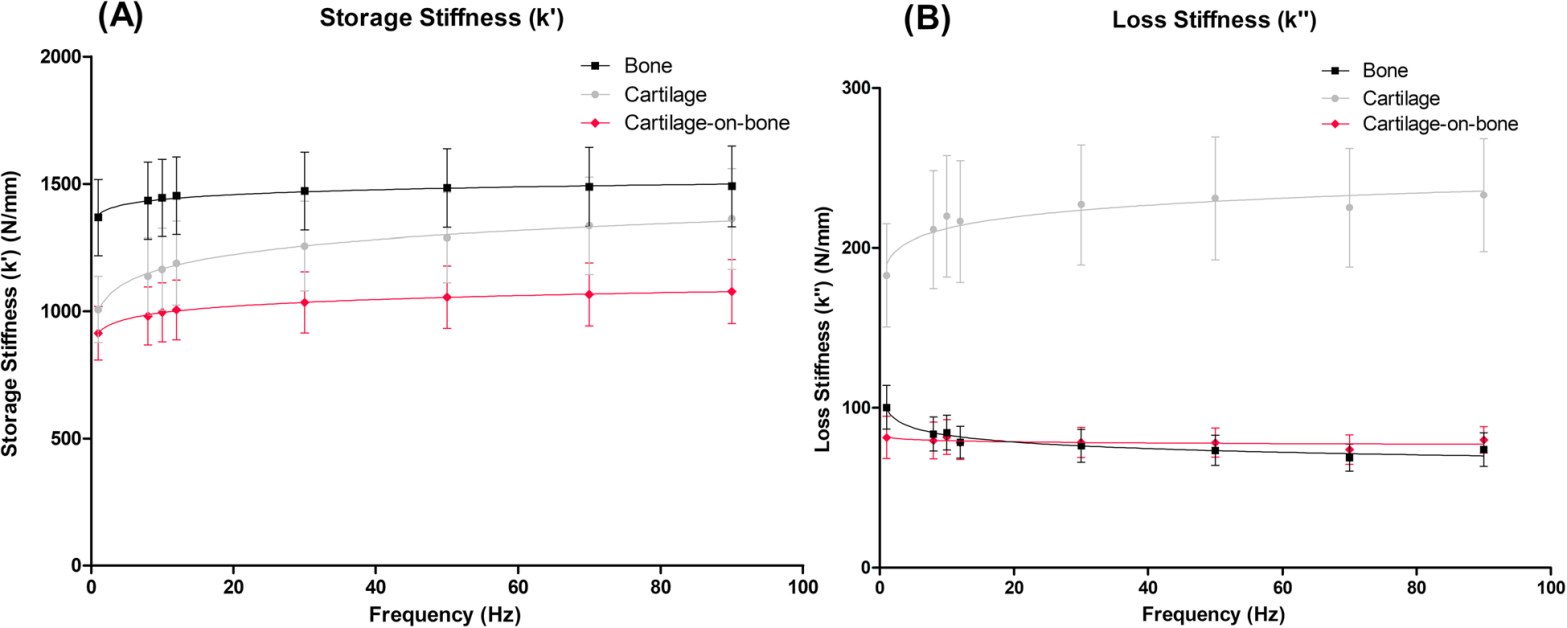
Storage (**a**) and loss (**b**) stiffness for the cartilage-bone-system, isolated cartilage, and isolated bone specimens plotted against frequency from 1 to 90 Hz (mean ± 95% confidence intervals, (*N* = 5) with natural logarithmic regression trendlines). In total, 19 cartilage-bone cores, 19 cartilage, and 18 bone specimens from five femoral heads were tested. Results are displayed on a linear scale

Cartilage-bone cores, cartilage, and bone specimens were found to be frequency-dependent with respect to *k’* (Eq. 7, Fig. 4a). Consistently, *k’* was observed to increase with frequency for all specimens, with the mean cartilage-bone specimen result as 1016 ± 54.0 N.mm^− 1^, cartilage 1217 ± 117 N.mm^− 1^, and bone 1455 ± 40.7 N.mm^− 1^. For *k’*, differences between all three specimen types (cartilage-bone core, cartilage, and bone), were found to be statistically significant (*p* < 0.001).

#### Loss stiffness (k”)

Loss stiffness was frequency-dependent for cartilage and bone specimens (Eq. 7) with a mean value of 218 ± 16.1 N.mm^− 1^ for cartilage and 79.8 ± 9.77 N.mm^− 1^ for bone. However, loss stiffness was found to be frequency-independent for the cartilage-bone-system (Fig. 4b). A significant difference (*p* < 0.001) was observed between *k”* values for the cartilage-bone core and cartilage specimens, as well as between isolated cartilage and bone specimens. However, there was no significant difference (*p* > 0.05) found for *k”* between the cartilage-bone-system and isolated bone specimens.

### Total energy dissipated

**Fig. 5:**
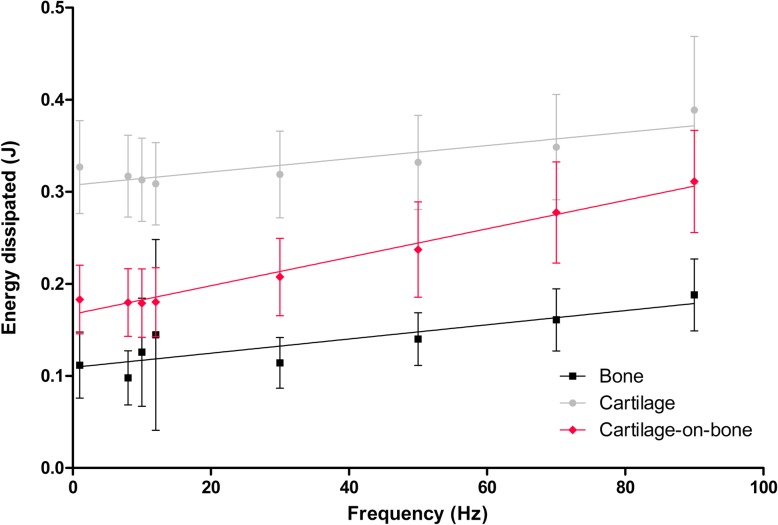
Total energy dissipated for cartilage-bone cores, cartilage, and bone specimens plotted against frequency from 1 to 90 Hz (mean ± 95% confidence intervals, (*N* = 5) with linear regression trendlines). In total, 19 cartilage-bone cores, 19 cartilage, and 18 bone specimens from five femoral heads were tested. Results are displayed on a linear scale

Energy dissipated was found to be frequency-dependent for all three specimen types (Eq. 8) with a mean value of 0.143 ± 0.101 J for bone specimens, 0.339 ± 0.111 J for cartilage specimens, and 0.2184 ± 0.094 J for cartilage-bone cores (Fig. 5). A significant difference (*p* < 0.05) was found between the total energy dissipated for all three groups.

8 $$ energy\ dissipated=A\ (f)+B\  for\ 1\le f\le 90\  Hz $$

### Histomorphological analysis

**Fig. 6:**
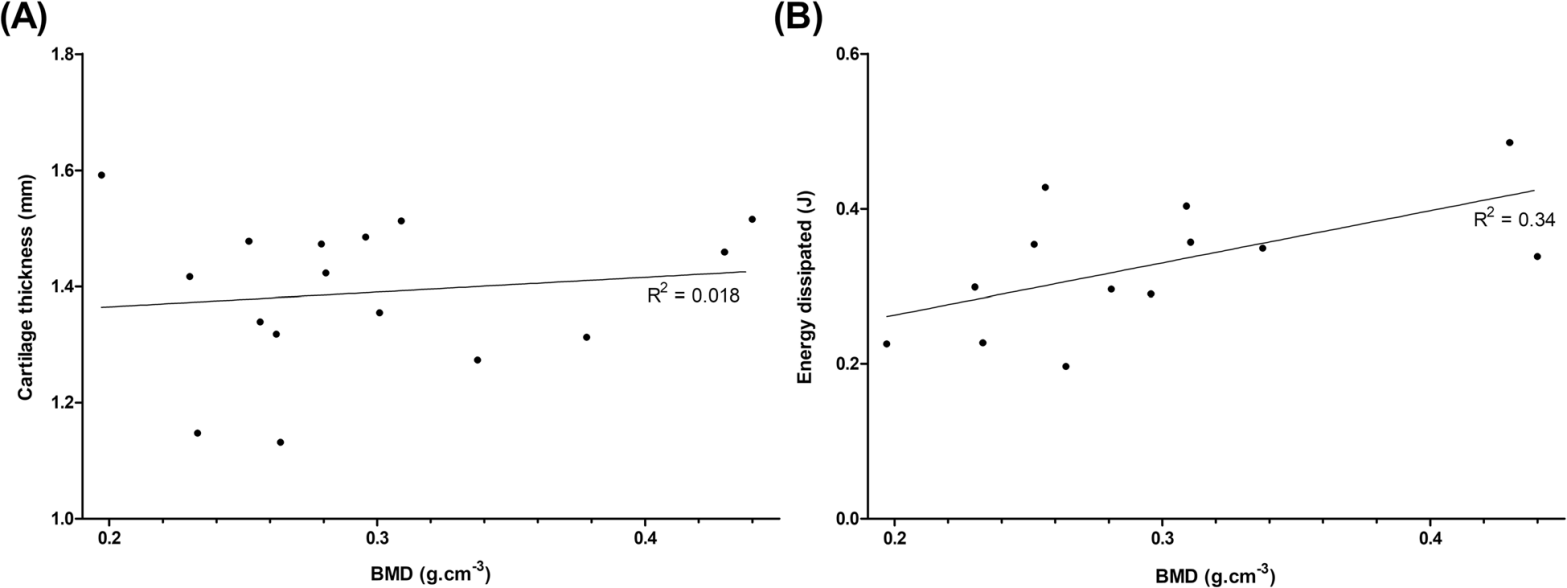
**a** Isolated cartilage thickness plotted against BMD (linear regression (solid line), (*N* = 5)). In total, 16 specimens from five femoral heads were tested. **b** Linear regression analysis of BMD and total energy dissipated from cartilage at 1 Hz (linear regression trendline, (*N* = 5)). In total, 13 specimens from five femoral heads were tested. Results displayed on a linear scale

The mean BMD (*ρ*) for all bone specimens tested was + 0.286 ± 0.081 g.cm^− 3^. Regression analysis of BMD and cartilage thickness values did not reveal any significant relationship (*p* = 0.62) (Fig. 6a). Linear regression analysis (Eq. 9) was performed on BMD (*ρ*) and mean total energy dissipated from cartilage specimens (Fig. 6b, Table 3). The relationship between these properties was found to be statistically significant (*p* < 0.05) at all frequencies except at 90 Hz (*p* = 0.09).

9 $$ Energy\ dissipated=A\ \left(\rho \right)+B $$

**Table 3:** R^2^ and *p*-values from linear regression analysis of BMD (*n* = 13) and total energy dissipated from isolated cartilage specimens (*n* = 13) for each frequency. Data displayed to 2 significant figures

Frequency (Hz)	*p*-value	R^2^
1	0.035	0.34
8	0.017	0.39
10	0.021	0.37
12	0.017	0.39
30	0.021	0.37
50	0.032	0.33
70	0.046	0.29
90	0.093	0.22

No significant relationships existed between BMD and the storage or loss moduli of the cartilage, or the storage and loss moduli of the bone. Furthermore, there were no significant relationships found between total energy dissipated and the thickness of the isolated cartilage, isolated bone, or the osteochondral specimens respectively.

## Discussion

This study established the viscoelastic behaviour of isolated cartilage and bone specimens in compression, and compared it with the osteochondral core, an analogy not previously conducted with human tissue. Cartilage-bone cores, isolated cartilage, and subchondral bone specimens were determined to be viscoelastic across all frequencies tested. Significant differences in behaviour of the core compared with the isolated specimens for *k’* and *k”* were identified. Most notably, cartilage-bone specimens displayed a frequency-independent trend for *k’* unlike isolated specimens, which were frequency-dependent. Previous research has focused on cartilage or cartilage-bone specimens alone [4, 6, 13, 18] and has not looked at the independent properties of human subchondral bone. DMA testing of human subchondral bone was conducted in the present work and the results highlight a significant difference in the viscoelastic properties of bone in comparison to cartilage; *E”* was shown to increase with increasing frequency for cartilage yet decrease with increasing frequency for bone. This data emphasises the complex mechanical properties of osteochondral tissues and in particular demonstrates the significance of the cartilage-bone interface.

A previous study by Temple et al. characterised the viscoelastic properties of human articular cartilage and revealed that this tissue behaves in a frequency-dependent manner, fitting a logarithmic function, thus supporting the findings of this study [4]. They reported storage and loss moduli for articular cartilage from human femoral heads to be 31.9–43.3 MPa and 5.3–8.5 MPa respectively. This is in line with reported mean storage and loss moduli in the present analysis of 34.4 ± 3.35 MPa and 6.17 ± 0.48 MPa respectively. In addition, Jeffrey & Aspden [35] calculated a ‘dynamic’ modulus for human articular cartilage from femoral heads by applying an impact load. The resulting dynamic modulus of human articular cartilage at stresses of 10 MPa and 23 MPa was reported to be 64 ± 13 MPa and 85.1 ± 4.9 MPa respectively. This is higher than the values reported for cartilage storage modulus in this study, although it is important to note that the induced stresses applied by Jeffrey & Aspden [35] were much higher, and higher stress is known to increase the storage modulus [13]. The present study has demonstrated that both storage and loss modulus for cartilage increase with increasing frequency. Notably, more energy is stored by both cartilage and bone tissues than is dissipated, with the effect most apparent at higher frequencies. It was determined that *E”* was an order of magnitude lower than *E’* for bone across all frequencies tested. Similarly, *E”* for cartilage was lower than *E’* between 1 and 90 Hz, as confirmed by other authors [4]. A small subset of the population is known to have high heel-strike rise times during gait, which induce forces at high frequencies (as high as 92 Hz) on the lower limbs [3]. Initially hypothesised by Radin et al. [36], high-frequency loading on the lower limbs has been identified as a marker of early onset osteoarthritis (OA), a degenerative disease that results in cartilage loss [37]. This study demonstrated increased energy storage for both cartilage and bone at higher frequencies, with energy dissipation determined to be an order of magnitude lower than energy storage. This behaviour could be a contributing factor to disease progression due to damaging stress concentrations. This is significant, as subchondral bone tissue in particular is likely to incur damage due to its increased ability to store energy yet decreased ability to dissipate it. However, as bone has a higher regenerative capacity than cartilage [38], this may be a mechanism for preventing cartilage damage, as the bone is more readily able to heal.

Bovine articular cartilage is considered a good model for human articular cartilage, as it displays similar trends in viscoelastic properties [4]. A recent study by Fell et al. [19] determined the viscoelastic properties of bovine cartilage and subchondral bone. They reported mean storage and loss moduli of bovine cartilage to be 45 MPa and 5.5 MPa respectively, whilst isolated bovine subchondral bone was found to have a mean storage modulus of 110 MPa and a mean loss modulus of 5 MPa. The storage modulus for bovine cartilage was therefore 1.3 times higher than isolated human cartilage in this study, which had a mean of 34.4 MPa. However, the bovine storage moduli for bone and loss moduli for cartilage reported by Fell et al. [19] were found to be lower than the respective results for the isolated human specimens in this study, though in the same order of magnitude. A possible reason for this is that the bovine tissues were obtained from the knee joint, whereas the human specimens were from the hip. Therefore, anatomical region is important when investigating and comparing the mechanical properties of osteochondral tissues.

Similar trends to the results in the present study were displayed by Fell et al. [19] for cartilage and bone storage and loss moduli (1–90 Hz). Of note, the loss modulus trends found in bovine tissue were also evident in human tissue (Fig. 3b). Whilst the trends were similar for bovine and human osteochondral tissue, Fell et al. evidenced a ‘crossover’ of the cartilage and bone loss modulus values [19]. At 1 Hz, bone had a higher loss modulus than cartilage, but at frequencies above 8 Hz this was reversed with cartilage samples exhibiting a higher loss modulus than bone. The authors suggested this might be a mechanism to prevent cartilage damage, as cartilage will dissipate more energy than bone under high-frequency loading. However, this trend was not noted in human osteochondral tissue, where bone had a higher loss modulus than cartilage at all frequencies tested (Fig. 3b). This data raises the hypothesis that cartilage damage in OA patients could be due to an inability of cartilage to dissipate energy into the bone under high-frequency loading, but further experimental work would be required to evidence this. A comparison of the trends seen here with Fell et al. demonstrates the importance of studying human osteochondral tissue since it is clear that animal models do not always display the same trends [4]. The present study is the first to report viscoelastic properties of human articular cartilage, subchondral bone, and the osteochondral core using Dynamic Mechanical Analysis.

As well as investigating isolated tissues, this research aimed to better understand the osteochondral core as a whole system. Storage stiffness for the cartilage-bone system was logarithmically frequency dependent and lower than cartilage and bone for all frequencies tested (Fig. 4). Loss stiffness for the cartilage-bone system was independent of frequency and lower than isolated specimens across the range of frequencies tested. These results are in line with previous work, which looked solely at bovine cartilage-bone cores and found loss stiffness to be frequency-independent [13, 18]. The difference in behaviour of cartilage isolated from and attached to subchondral bone is emphasised here and has demonstrated that cartilage should not be considered in isolation when determining properties representative of in vivo behaviour. The data obtained in our study supports the development and testing of whole tissue-replacement systems as opposed to cartilage replacement materials in isolation.

Prior studies of bovine cartilage both on- and off-bone found the loss modulus of on-bone cartilage to be frequency-independent, whereas cartilage off-bone has a frequency-dependent modulus [13, 18]. Lawless et al. [13] found that there was no dependency of the storage stiffness on the presence or absence of the underlying subchondral bone, and therefore proposed that on-bone cartilage may be more predisposed to failure than off-bone cartilage due to the storage/loss ratio being higher for cartilage on-bone. The findings of the present study report the same frequency-independence for cartilage on-bone loss modulus, with isolated cartilage displaying a frequency-dependent trend. Thus, findings reported in prior studies support the current results, although it should be noted that the aim of the present study was focused on the viscoelasticity of subchondral bone and the role of the cartilage-bone interface, rather than the cartilage itself. Hence, a more detailed discussion on the viscoelastic properties of cartilage both on- and off-bone is provided elsewhere [13].

In order to explain the difference in behaviour between isolated cartilage and the cartilage-bone-system system, Edelsten et al. [12] suggested that cartilage attached to subchondral bone is more constrained in its deformation, and this may lead to it appearing stiffer and more elastic than when in isolation. Experimentally, this result has been verified as *k”* becomes frequency-independent under load [12, 13]. However, the trends in *E’* and *E”* in isolated bone identified in this study (Fig. 3) may infer an additional explanation. Bone was found to be positively frequency-dependent for storage and negatively frequency-dependent for loss moduli. Therefore, the constraining effects of the bone could not be the sole reason for the frequency independence of *k”* for the cartilage-bone-system. A decrease in loss stiffness indicates a decrease in the energy dissipated, suggesting that bone does not dissipate as much energy to the surrounding tissue at higher frequencies. This may prevent the load energy being returned to the cartilage, which could be a further mechanism to prevent cartilage damage. While observing equine osteochondral cores under high-impact, Malekipour et al. identified that bone can absorb a much higher amount of impact energy than cartilage [39]. Furthermore, it is often bone that breaks during high-impact loading, as the main mechanism by which it absorbs energy is through trabeculae fracture [39]. Although this may be desirable, as bone has a greater propensity to heal than cartilage, this may put the joint at risk of long-term damage. Clearly, the interface between the two tissues plays an essential role in transferring the load energy through the cartilage and into the bone.

A significant difference was identified between loss stiffness values for the cartilage-bone system and cartilage specimens. In contrast, when comparing the cartilage-bone system and bone specimens this was not observed, signifying that the loss stiffness of the osteochondral core is more closely aligned to the loss stiffness of bone than of isolated cartilage. This suggests a key property to consider is the energy transfer, including dissipation of energy, through the osteochondral junction. In order to characterise this in the present study, the total energy dissipated from the cartilage at each frequency during DMA was calculated. The benefit of this approach is the entire system is considered as a whole by determining absolute values rather than material properties. The results demonstrated that cartilage dissipated higher total energy than bone and the cartilage-bone system across all frequencies tested (Fig. 5). While expected, as one of the key roles of articular cartilage is to dissipate energy into the underlying bone, to the best of the authors knowledge this is the first report of such a relationship from human tissue analysis.

It has been hypothesised that high heel-strike rise times during gait causes a predisposition to the onset of OA [6, 36]. This high-impact loading, combined with a reduced ability to dissipate energy at high frequencies, is likely to cause damage to the bone, leading to subchondral bone remodelling and therefore a higher BMD. Prior to work by Fell et al. [19], no previous studies had attempted to link bone histomorphology with viscoelastic properties of osteochondral tissues. The study on bovine osteochondral cores identified a significant relationship between subchondral bone BMD and cartilage loss stiffness [19]. Correlations between cartilage thickness with BMD and subchondral bone plate thickness were also unearthed. This suggests that cartilage health is interrelated with the histomorphological properties of the subchondral bone. The results of the present study demonstrated a statistically significant (*p* < 0.05) link at all frequencies except at 90 Hz (*p* = 0.09) between the total energy dissipated by isolated cartilage specimens and BMD (Fig. 6b). This supports the findings by Fell et al. and validates the hypothesis that the dissipation of energy through to the subchondral bone provides mechanical signals, which can alter the structure of the tissue, for example a remodelling of the trabeculae [40]. It should be noted that although the goodness of fit is low between the BMD and total energy dissipated from the cartilage, they are comparable to R and R^2^ values presented in other BMD studies [19, 41, 42].

There are a small number of limitations within the present study. One of the key challenges with human tissue is that specimens can have wide variation due to factors such as age, weight, and gender, which are known to have an effect on the health of these tissues. In addition, the patients who donated their tissue for this study are of an advanced age and therefore may not be representative of younger adults. Furthermore, it has been previously reported that bovine cartilage stiffens with age [43]. Whilst the tissues in the present study are obtained from older adults, the joints showed no sign of OA joint damage upon chondropathy assessment and are representative of cartilage that has maintained health throughout its lifespan. The high variation between specimens, combined with reduced specimen numbers due to tissue availability, is likely to be the reason that a significant link between BMD and cartilage viscoelasticity was not established in the present study, despite being demonstrated in bovine tissue [19]. It should also be noted that the cartilage surface in the sample blocks exhibited a slight curvature (Fig. 1b). To minimise the impact of this on mechanical test data, cores were taken from flatter regions of the block, and hence it was not accounted for. There are advantages and disadvantages to using stiffness measurements, as reported in this study. The calculation of storage and loss moduli is dependent on thickness, and therefore any limitations associated with thickness measurements are included in moduli, but are not included in stiffness [44], however, direct comparison of stiffness can only be made for the same shape and size of specimens. As moduli and stiffness are fundamentally different measurements, it limits the comparability across literature, although their trends with frequency of loading can be compared because the modulus is simply the stiffness divided by a constant (i.e. a shape factor) [13]. The primary reason for choosing to report stiffness in this study is that the osteochondral cores were a structure consisting of both soft and hard tissues. A separate limitation during DMA testing of biological tissues is damping if these are tested in a fluid medium. To avoid potential damping due to fluid in this study, the samples were not submerged in fluid and, therefore, the dissipation of energy reported was solely due to the cartilage tissue. Damping has been previously shown to have little effect between samples tested in air and in Ringer’s solution [13].

The complexity of the frequency-dependent viscoelastic properties of cartilage and bone described in this study demonstrate that there is a sophisticated interaction between these two tissues in regard to storage and dissipation of energy. High-frequency loading results in an increased storage of energy in the subchondral bone, a likely mechanism to prevent cartilage damage, which is a factor that should be further investigated in relation to the progression of OA. The results obtained in this study provide details of native tissue behaviour in vitro at physiologically relevant frequency ranges, the first reported values in human osteochondral tissue under dynamic loading.

## Conclusions

This study demonstrates that subchondral bone is viscoelastic over a physiological frequency range (1–90 Hz). Storage and loss moduli for cartilage and storage modulus for bone increase with increasing frequency, while the loss modulus of bone decreases. Loss stiffness for the cartilage-bone-system is frequency-independent yet for isolated specimens it was shown to be frequency-dependent. Finally, a statistically significant link has been identified between cartilage energy dissipated and bone histomorphology. From these results, the importance of characterising the properties of articular cartilage both isolated from, and attached to, subchondral bone is clearly highlighted. Overall, this work demonstrates the significance of the bone-cartilage interface, in particular for energy storage and dissipation.

## Data Availability

The datasets used and/or analysed during the current study are available from the corresponding author on reasonable request.

## Abbreviations

BMD: Bone mineral density
CaHA: Calcium hydroxylapatite
DMA: Dynamic mechanical analysis
OA: Osteoarthritis
μ-CT: Micro-computed tomography
